# Supplementary material for: Computing the relative binding affinity of ligands based on a pairwise binding comparison network
Source: Nat Comput Sci. 2023 Oct 19;3(10):860–72. doi: 10.1038/s43588-023-00529-9 (PMC10766524; doi:10.1038/s43588-023-00529-9)
Supplement: Supplementary file 1 — Supplementary Section 1, Figs. 1–4 and Tables 1–10. [file 43588_2023_529_MOESM1_ESM.pdf]

nature computational science

Article

https://doi.org/10.1038/s43588-023-00529-9

Computing the relative binding affinity of ligands based on a pairwise binding comparison network

In the format provided by the authors and unedited

Content

Supplementary Sections 2

Supplementary Section 1 2

Supplementary Figures 3

Supplementary Figure 1 3

Supplementary Figure 2 4

Supplementary Figure 3 5

Supplementary Figure 4 6

Supplementary Tables 7

Supplementary Table 1 7

Supplementary Table 2 8

Supplementary Table 3 9

Supplementary Table 4 10

Supplementary Table 5 11

Supplementary Table 6 12

Supplementary Table 7 13

Supplementary Table 8 14

Supplementary Table 9 15

Supplementary Table 10 16

Supplementary Sections

Supplementary Section 1. The robust performance of PBCNet to binding poses changes

Docking poses are often influenced by factors such as docking software, docking methods, and pose selection process. As a result, the docking poses obtained may not perfectly reproduce the crystal structures. Therefore, studying the effect of small changes in ligand poses on the predictive ability of PBCNet is highly relevant in real-world applications. In this study, we explore this issue with the FEP1 set.

First, for each PDB used in the FEP1 set, we redocked co-crystallized ligands to their corresponding proteins using Glide SP with default parameters. The docking poses with RMSDs less than 1.0 Å were retained as biased poses, which are all slightly different from the experimentally determined poses. Then, the biased poses of the remaining compounds were generated for each system through the shape-constrained docking process (Schrödinger 2020-4), where the biased poses of the co-crystallized ligand were sequentially used as shape references. Through these operations, we constructed the biased FEP1 set (Supplementary Table 9), where each system has several biased poses.

To evaluate the ranking performance of PBCNet on the biased FEP1 set, we used the same protocol as described in '*Zero-shot learning*' section of the main text, and the mean values and variances of the ranking metrics are summarized in Supplementary Table 10. It can be found that the ranking performance of PBCNet on the biased FEP1 set is very similar to that on the original FEP1 set (Pearson: 0.64 vs 0.65, Spearman: 0.64 vs 0.64). This result clearly demonstrated that our model is highly robust to small changes in ligand poses. One potential reason for this robustness is that the ligand poses used for model training were produced by molecular docking. Some variance has been introduced in the binding pose generation procedures, which may be considered as a data augmentation.

Supplementary Figures

Supplementary Figure 1

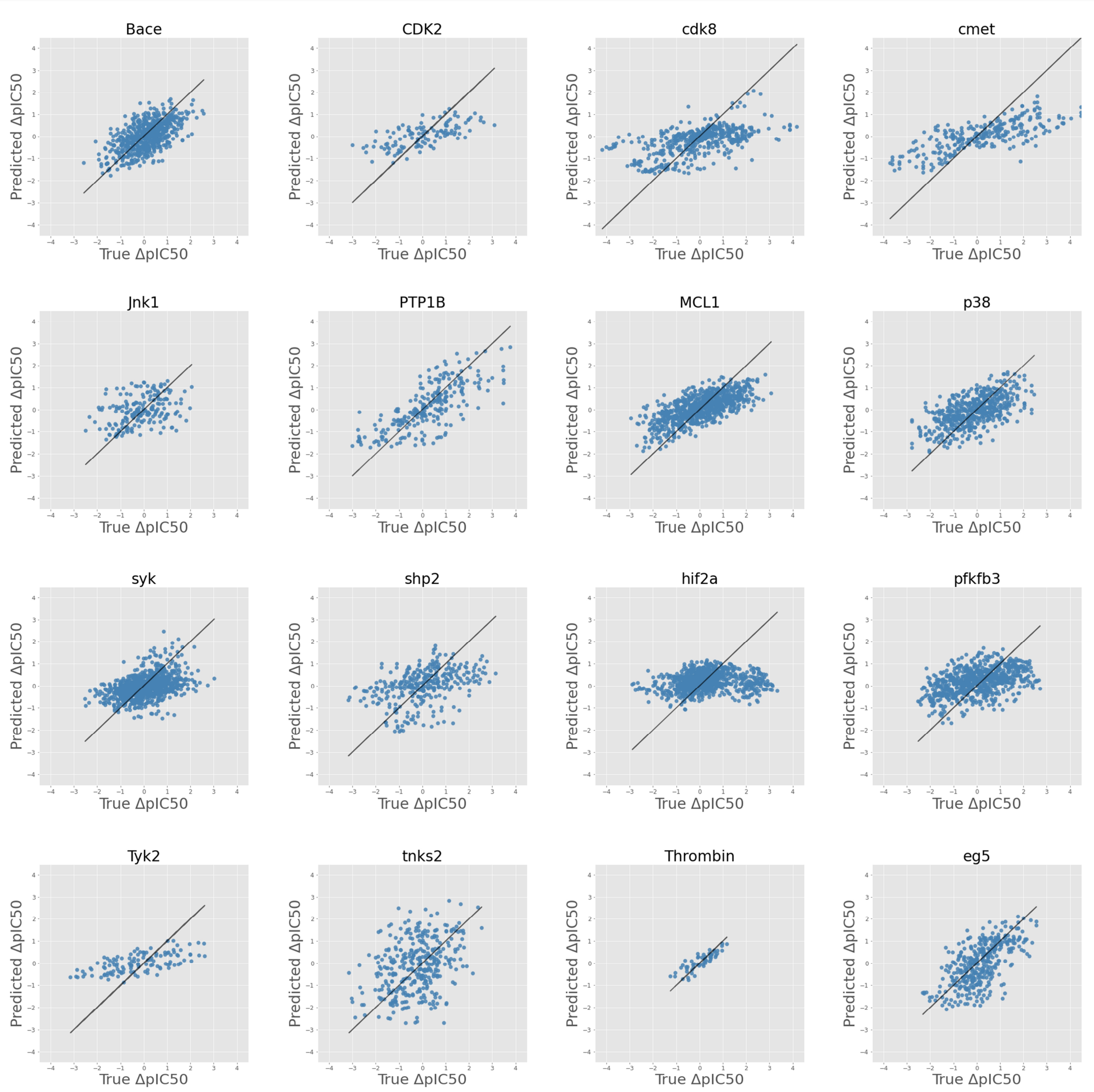

Supplementary Figure 1. The scatter diagram of the prediction results of PBCNet on FEP1 and FEP2 sets. The y-axis indicates the predicted ΔpIC_{50} values and the x-axis indicates the true ΔpIC_{50} values. For an ideal model the dots should be distributed around the diagonal line.

Supplementary Figure 2

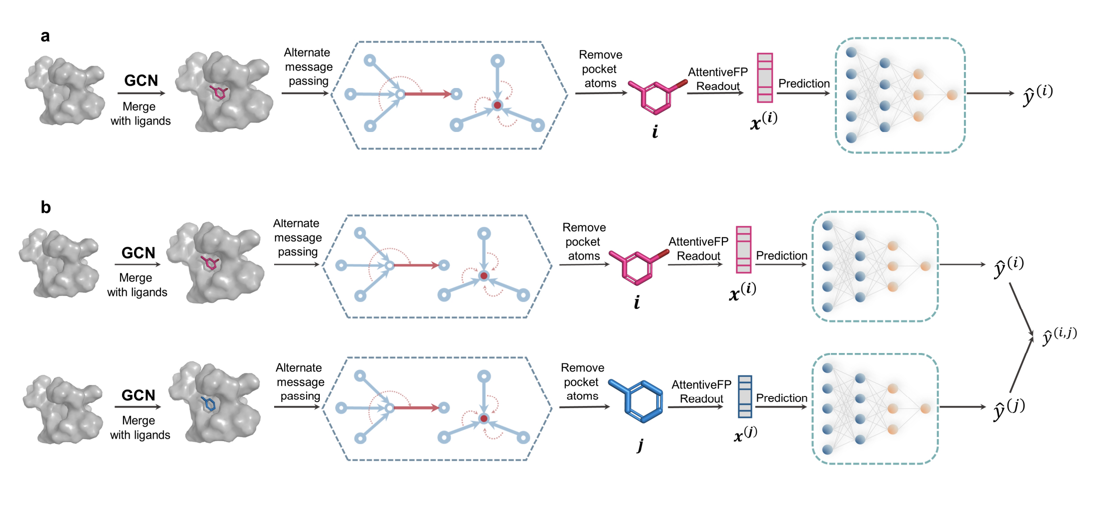

Supplementary Figure 2. Schematic of the framework of the model used in the ablation experiments. **a.** The frameworks of Singular PBCNet. The model directly predicts the absolute activity values of compounds, and the red node indicates the graph level representation of the red ligand. **b.** The frameworks of Separated PBCNet. The model first predicts the absolute binding free energies of two inputted compounds, and then directly uses the difference of the predicted absolute binding free energies as the final relative binding free energies prediction for loss calculation. The red and blue nodes indicate the graph level representation of the red and blue ligands.

Supplementary Figure 3

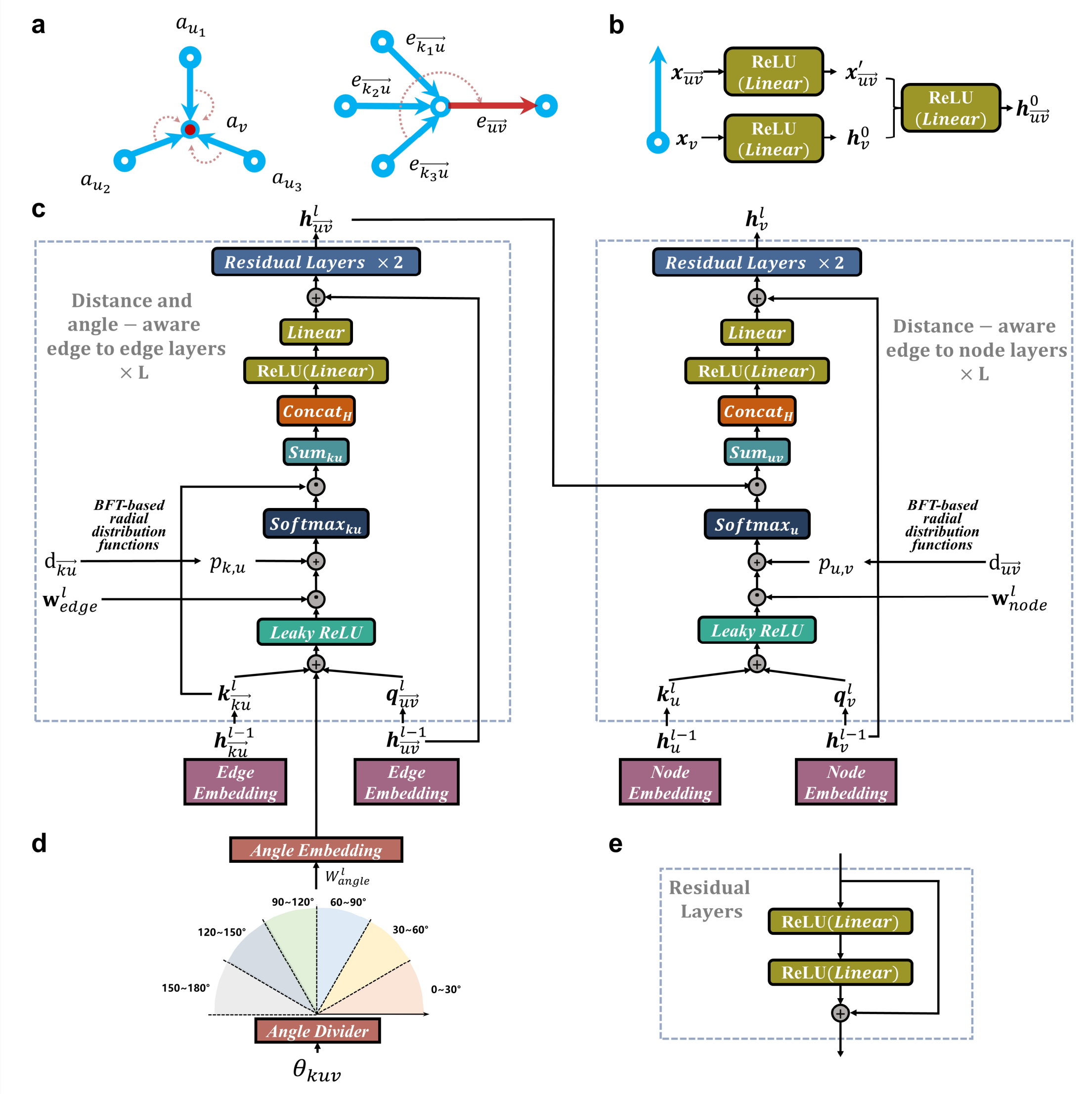

Supplementary Figure 3. Schematic diagram of AU-MPNN correlation algorithm. **a.** Schematic diagram of neighbor nodes and neighbor edges; $\{a_{u_1}, a_{u_2}, a_{u_3}\}$ are neighbor nodes of a_v, and $\{e_{\overrightarrow{k_1u}}, e_{\overrightarrow{k_2u}}, e_{\overrightarrow{k_3u}}\}$ are neighbor edges of $e_{\overrightarrow{uv}}$. **b.** The calculation process diagram of the initial hidden representations of a_v and $e_{\overrightarrow{uv}}$ ($\boldsymbol{h}_v^0$ and $\boldsymbol{h}_{\overrightarrow{uv}}^0$); $\boldsymbol{x}_v$ and $\boldsymbol{x}_{\overrightarrow{uv}}$ are their initial features, and $\boldsymbol{x}'_{\overrightarrow{uv}}$ is an intermediate vector to obtain $\boldsymbol{h}_{\overrightarrow{uv}}^0$. **c.** The specific architecture of AU-MPNN; the left is the structure of distance and angle-aware edge to edge (DAEE) blocks and the right is the structure of distance-aware edge to node (DEN) blocks. **d.** The calculation process diagram of angle embedding, which can also be seen as a part of the DAEE block. **e.** Schematic diagram of residual layers.

Supplementary Figure 4

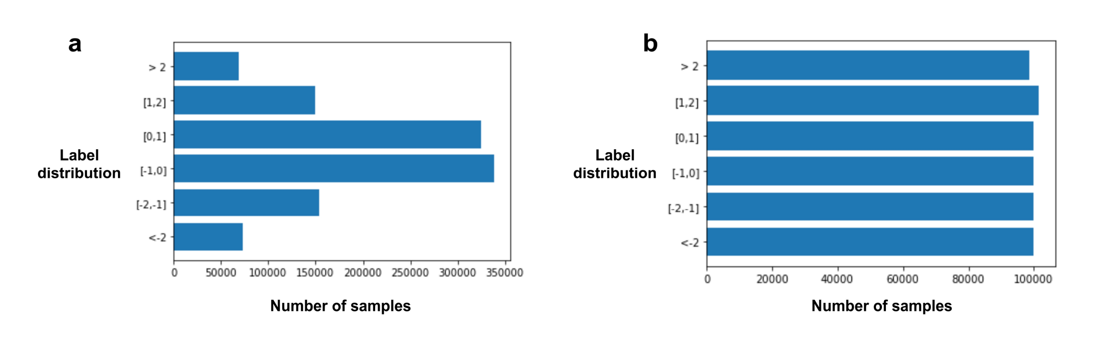

Supplementary Figure 4. Statistical charts of data distribution**. a.** The label distribution of the original training dataset. **b.** The label distribution of the balanced training dataset. The y-axis denotes the interval to which the training data labels belong and the x-axis denotes the number of training data. We resampled the training data from a normal distribution to a uniform distribution to reduce the risk of model overfitting

Supplementary Tables

Supplementary Table 1

Test sets	Metrics	PBCNet finetuned with 2 ligands (new testing series)	PBCNet finetuned with 2 ligands (original testing series)	PBCNet finetuned with 6 ligands (new testing series)	PBCNet finetuned with 6 ligands (original testing series)	PBCNet finetuned with 10 ligands (new testing series)	PBCNet finetuned with 10 ligands (original testing series)	PBCNet without finetuning	FEP+
FEP1 set	R	0.663 (0.009)	0.669 (0.009)	0.729 (0.009)	0.729 (0.006)	0.731 (0.008)	0.737 (0.005)	0.648 (0.001)	0.74
	ρ	0.679 (0.009)	0.687 (0.011)	0.742 (0.012)	0.749 (0.007)	0.746 (0.009)	0.761 (0.005)	0.636 (0.002)	0.72
	$RMSE_{pw}$ ($kcal \cdot mol^{-1}$)	1.399 (0.118)	1.376 (0.111)	1.106 (0.058)	1.078 (0.061)	1.116 (0.081)	1.074 (0.083)	1.114	1.08
FEP2 set	R	0.527 (0.031)	0.527 (0.031)	0.592 (0.031)	0.595 (0.028)	0.646 (0.029)	0.646 (0.029)	0.468 (0.003)	0.66
	ρ	0.575 (0.022)	0.577 (0.022)	0.629 (0.020)	0.632 (0.020)	0.657 (0.025)	0.663 (0.019)	0.513 (0.002)	0.65
	$RMSE_{pw}$ ($kcal \cdot mol^{-1}$)	1.536 (0.124)	1.503 (0.142)	1.383 (0.088)	1.351 (0.087)	1.296 (0.078)	1.268 (0.086)	1.490	1.67

Supplementary Table 1. The performance of PBCNet with few-shot learning on the new and old testing series. The first column of the table denotes the different test sets and the second column denotes the different metrics, where R denotes Pearson's correlation coefficient, ρ denotes Spearman's rank correlation coefficient, and $RMSE_{pw}$ denotes the pairwise root-mean-square-error. One thing to keep in mind is that there are only 11 ligands in Thrombin (a testing series in FEP1 set), so the performance of the FEP1 set reported in the 7th column is based on the remaining 7 series. Numbers in parentheses indicate the variance of the results of the 10 independent runs (*n*=10).

Supplementary Table 2

System	Number of ligands	Experimental order	MM-GB/SA order	Advantage order	Advantage ratio	Efficiency improvement ratio
FGFR2	15	7	4	3	20.0%	75.0%
BCL6	25	23	1	22	88.0%	2200.0%
HO1	19	8	9	-1	-5.26%	11.0%
LRRK2	20	6	14	-8	-40.0%	57.0%
sEH	51	43	11	32	62.75%	291.0%
CKD9	38	13	17	-4	-10.53%	24.0%
WDR5	21	16	18	-2	-9.52%	11.0%
AAK1	30	28	28	0	0.0%	0.0%
PSK13	38	27	16	11	28.95%	69.0%
mean	28.56	19	13.11	5.89	14.93%	281.0%

Supplementary Table 2. Selection experiment results of the MM-GB/SA for 9 different datasets. The first column of the table indicates the name of the system, the second column is the number of compounds per system, the third column indicates the order of experimental synthesis of the target ligands (the ligand with the highest affinity in each chemical series), the fourth column indicates the order of selection of the target compounds for MM-GB/SA, and for the definition of the last three indicators, please refer to equation 1-3 in the main text.

Supplementary Table 3

Ligand name	Interaction type	Ligand atom	Protein atom	Protein atom (Remove hydrogen atom)
1a (Thrombin)	H-bond	O 3	H: GLY216	N: GLY216
	H-bond	N 8	O: SER214	-
	H-bond	N 10	O: GLY216	-
18660-1 (JNK1)	H-bond	N 12	O:GLU109	-
	H-bond	O 18	H:MET111	N:MET111
	Negative	C 5	-	-

Supplementary Table 3. Calculated intermolecular interactions of compounds 6a and 18660-1 with the corresponding proteins. The interaction type 'H-bond' indicates a hydrogen bond, and 'Negative' indicates that no intermolecular interaction was formed and is a negative control. The third column indicates the types of ligand atoms that formed the interaction and their numbers. The fourth column indicates the types of protein atoms that form interactions and their numbers. Since we did not consider hydrogen atoms, we chose to analyze heavy atoms covalently linked to hydrogen atoms when they were involved, and the results are listed in the last column.

Supplementary Table 4

Ligand name	Structure	Predicted pIC$_{50}$	Attribution	Attribution_N
1a (Reference ligand)	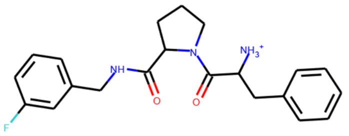	-	-	-
6a	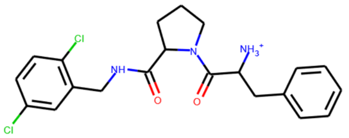	5.64	-	-
Sub$_0$	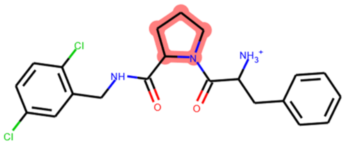	5.20	0.44	0.133
Sub$_1$	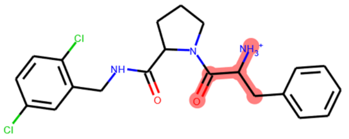	4.90	0.74	0.223
Sub$_2$	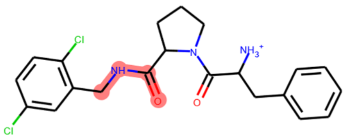	5.42	0.22	0.066
Sub$_3$	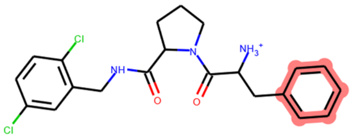	5.27	0.37	0.111
Sub$_4$	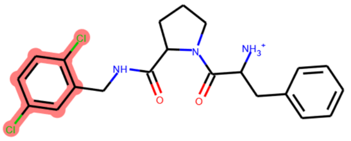	4.09	1.55	0.467

Supplementary Table 4. Structure and predict activity relationship of a substructure-masked Thrombin inhibitor. The first column indicates the name of the compound, where 1a is the reference molecule, the second column is the structure of the compound, the highlighted part indicates the masked sub-structures. The definition of the last two indicators, please refer to equation 4 and 5 in the main text.

Supplementary Table 5

Model	FEP1	FEP2	Mean
Singular PBCNet	0.559 (0.0781)	<u>0.372 (0.0390)</u>	<u>0.465 (0.0345)</u>
Separated PBCNet	0.632 (0.945)	<u>0.425 (0.0390)</u>	0.528 (0.231)
MSE PBCNet	0.629 (0.547)	0.488 (0.148)	0.559 (0.105)
PBCNet (without aromatic information)	0.627 (0.547)	0.478 (0.383)	0.553 (0.323)
PBCNet (without distance information)	0.586 (0.250)	<u>0.433 (0.0391)</u>	<u>0.510 (0.0131)</u>
PBCNet (without angle information)	0.631 (0.945)	0.502 (0.945)	0.567 (0.940)
PBCNet	**0.636**	**0.513**	**0.575**

Supplementary Table 5. The results of ablation experiment. The first column indicates the name of the ablation models. Spearman's rank correlation coefficient is used as the evaluation metric. Best results are highlighted in boldface type. Numbers in parentheses indicate statistical significance test values, and those with statistically significant differences (p-values less than 0.05) are underlined. Statistical significance was calculated using the two-side Wilcoxon signed rank tests, where Singular PBCNet and Separated PBCNet were calculated with MSE PBCNet and other models were calculated with PBCNet.

Supplementary Table 6

Atom Feature	Size	Description
Atom type	9	[C, N, O, F, P, S, Cl, Br, other]
Degree	6	Number of covalent bonds<br>[0, 1, 2, 3, 4, 5]
Formal charge	5	Integer electronic charge assigned to atom<br>[-2, -1, 1, 2, 0]
Chirality	4	[unspecified, tetrahedral CW, tetrahedral CCW, other]
Hydrogens	5	Number of connected hydrogens<br>[0, 1, 2, 3, 4]
Hybridization	5	[sp, sp^2, sp^3, sp^3d, sp^3d^2]
Periods	6	The row number of the periodic table of elements<br>[1, 2, 3, 4, 5, 6]
Groups	18	The column number of the periodic table of elements<br>[1, 2, 3, 4, 5, 6, 7, 8, 9, 10, 11, 12, 13, 14, 15, 16, 17, 18]
Atomic mass	1	Mass of the atom, divided by 100
Explicit valence	1	Explicit valence of the atom, divided by 10
Implicit valence	1	Implicit valence of the atom, divided by 10
Vdw radius	1	van der Waals radius of the atom
Aromaticity	1	Whether this atom is part of an aromatic system
Is acceptor	1	Whether this atom is an acceptor of hydrogen bonds
Is donor	1	Whether this atom is a donor of hydrogen bonds

Supplementary Table 6. Atomic features. The first column indicates the feature name and the second column indicates the feature dimension.

Supplementary Table 7

Bond Feature	Size	Description
Bond type	4	[single, double, triple, aromatic]
Conjugated	1	Whether the bond is conjugated
In ring	1	Whether the bond is part of a ring
Stereo	6	[none, any, E, Z, cis, trans]
Is covalent bond	1	Whether this bond is a covalent bond or a virtual bond

Supplementary Table 7. Bond features. The first column indicates the feature name and the second column indicates the feature dimension.

Supplementary Table 8

Protein	Number of ligands	PDB ID
FGFR2	15	7OZY
BCL6	25	7Q7R
HO1	19	3TGM
LRRK2	20	7SUF
sEH	51	7P4K
CKD9	38	7NWK
WDR5	21	7U9Y
AAK1	30	7RJ7
PSK13	38	5V3Y

Supplementary Table 8. The information of the benchmark dataset for simulation-based experiment. The first column indicates the system name, the second column indicates the number of compounds, and the last column indicates the PDB ID of the protein used here.

Supplementary Table 9

Systems	Number of biased series	RMSDs
BACE	7	0.189 Å, 0.218 Å, 0.242 Å, 0.27 Å, 0.278 Å, 0.290 Å,0.372 Å
CDK2	3	0.229 Å, 0.354 Å, 0.5 Å
JNK1	3	0.422 Å, 0.499 Å, 0.535 Å
MCL1	5	0.108 Å, 0.356 Å, 0.425 Å, 0.568 Å, 0.597 Å
p38	3	0.544 Å, 0.835 Å, 0.768 Å
PTP1B	7	0.627 Å, 0.641 Å, 0.7 Å, 0.708 Å, 0.710 Å, 0.774 Å, 0.861 Å
Thrombin	3	0.191 Å, 0.255 Å, 0.453 Å
Tyk2	6	0.146 Å, 0.22 Å, 0.622 Å,0.658 Å, 0.736 Å,0.96 Å

Supplementary Table 9. The statistical table of the biased poses of each system in FEP1 set. The first column indicates the name of the system, the second column indicates the number of biased poses, and the last column indicates the RMSD (Root Mean Square Deviation) between the biased pose of the co-crystallized ligand and its true pose.

Supplementary Table 10

		BACE	CDK2	JNK1	MCL1	p38	PTP1B	Thrombin	Tyk2	Average
Original poses	Pearson	0.61	0.66	0.41	0.72	0.56	0.75	0.84	0.64	0.65
	Spearman	0.52	0.66	0.47	0.73	0.56	0.71	0.82	0.61	0.64
Biased poses (Poses with deviations)	Pearson	0.61 ($1.1*10^{-4}$)	0.69 ($8.8*10^{-5}$)	0.43 ($2.9*10^{-5}$)	0.66 ($3.0*10^{-4}$)	0.61 ($2.6*10^{-4}$)	0.73 ($8.8*10^{-4}$)	0.83 ($5.3*10^{-5}$)	0.55 ($2.0*10^{-3}$)	0.64
	Spearman	0.58 ($1.0*10^{-3}$)	0.69 ($1.6*10^{-4}$)	0.48 ($2.1*10^{-4}$)	0.69 ($3.2*10^{-4}$)	0.62 ($6.5*10^{-4}$)	0.74 ($6.2*10^{-4}$)	0.84 ($8.2*10^{-5}$)	0.51 ($1.9*10^{-3}$)	0.64

Supplementary Table 10. The ranking performance of PBCNet on the original and biased FEP1 sets. The second column denotes the different metrics, where ‘Pearson’ denotes Pearson’s correlation coefficient, and ‘Spearman’ denotes Spearman’s rank correlation coefficient. For Biased poses, the mean and the variance (in brackets) of the ranking metrics are all reported (Bace: n=7, CDK2: n=3, JNK1: n=3, MCL1: n=5, p38: n=3, PTP1B: n=7, Thrombin: n=3, Tyk2: n=6).
