# Supplementary material for: Computing the relative binding affinity of ligands based on a pairwise binding comparison network
Source: Nat Comput Sci. 2023 Oct 19;3(10):860–72. doi: 10.1038/s43588-023-00529-9 (PMC10766524; doi:10.1038/s43588-023-00529-9)
Supplement: Supplementary file 2 — Reporting Summary [file 43588_2023_529_MOESM2_ESM.pdf]

nature portfolio

Corresponding author(s): Mingyue Zheng, Xiaomin Luo, Xutong Li

Last updated by author(s): Aug 29, 2023

Reporting Summary

Nature Portfolio wishes to improve the reproducibility of the work that we publish. This form provides structure for consistency and transparency in reporting. For further information on Nature Portfolio policies, see our Editorial Policies and the Editorial Policy Checklist.

Statistics

For all statistical analyses, confirm that the following items are present in the figure legend, table legend, main text, or Methods section.

n/a	Confirmed	
☐	☒	The exact sample size (*n*) for each experimental group/condition, given as a discrete number and unit of measurement
☐	☒	A statement on whether measurements were taken from distinct samples or whether the same sample was measured repeatedly
☐	☒	The statistical test(s) used AND whether they are one- or two-sided<br>*Only common tests should be described solely by name; describe more complex techniques in the Methods section.*
☐	☒	A description of all covariates tested
☒	☐	A description of any assumptions or corrections, such as tests of normality and adjustment for multiple comparisons
☐	☒	A full description of the statistical parameters including central tendency (e.g. means) or other basic estimates (e.g. regression coefficient) AND variation (e.g. standard deviation) or associated estimates of uncertainty (e.g. confidence intervals)
☐	☒	For null hypothesis testing, the test statistic (e.g. *F*, *t*, *r*) with confidence intervals, effect sizes, degrees of freedom and *P* value noted<br>*Give P values as exact values whenever suitable.*
☒	☐	For Bayesian analysis, information on the choice of priors and Markov chain Monte Carlo settings
☒	☐	For hierarchical and complex designs, identification of the appropriate level for tests and full reporting of outcomes
☐	☒	Estimates of effect sizes (e.g. Cohen's *d*, Pearson's *r*), indicating how they were calculated

Our web collection on statistics for biologists contains articles on many of the points above.

Software and code

Policy information about availability of computer code

Data collection	Python 3.7, Rdkit 2021.03.02, Schrödinger2020-4, pandas v1.1.5, numpy 1.23.5, scikit-learn 1.0.2
Data analysis	python 3.7, Rdkit 2021.03.02, pandas v1.1.5, numpy 1.23.5, scikit-learn 1.0.2, matplotlib 3.3.4

For manuscripts utilizing custom algorithms or software that are central to the research but not yet described in published literature, software must be made available to editors and reviewers. We strongly encourage code deposition in a community repository (e.g. GitHub). See the Nature Portfolio guidelines for submitting code & software for further information.

Data

Policy information about availability of data

All manuscripts must include a data availability statement. This statement should provide the following information, where applicable:

- Accession codes, unique identifiers, or web links for publicly available datasets
- A description of any restrictions on data availability
- For clinical datasets or third party data, please ensure that the statement adheres to our policy

The unprocessed training data is from BindingDB source and can be found at https://www.bindingdb.org/validation_sets/index.jsp. The test datasets used in this study are available at https://doi.org/10.5281/zenodo.8275244, where all molecule and protein files of FEP1 and FEP2 sets could be found. For benchmark dataset of the simulation-based experiment, all molecules and protein files also can be found at https://doi.org/10.5281/zenodo.8275244, including the following PDB files: 7OZY, 7Q7R, 3TGM, 7SUF, 7P4K, 7NWK, 7U9Y, 7RJ7, and 5V3Y. Source data for Fig. 2-4 and Fig. 5b is available with this manuscript.

Human research participants

Policy information about studies involving human research participants and Sex and Gender in Research.

Reporting on sex and gender	n/a
Population characteristics	n/a
Recruitment	n/a
Ethics oversight	n/a

Note that full information on the approval of the study protocol must also be provided in the manuscript.

Field-specific reporting

Please select the one below that is the best fit for your research. If you are not sure, read the appropriate sections before making your selection.

☒ Life sciences ☐ Behavioural & social sciences ☐ Ecological, evolutionary & environmental sciences

For a reference copy of the document with all sections, see nature.com/documents/nr-reporting-summary-flat.pdf

Life sciences study design

All studies must disclose on these points even when the disclosure is negative.

Sample size

1) In this study, the BindingDB protein-ligand validation sets (2020 version) were selected as the original training data source. A total of 1265 congeneric series were included in the dataset, and, for each series, SMILES (Simplified Molecular Input Line Entry System) of the ligands, PDB IDs of the available cocrystal structures, and corresponding binding affinity values were provided by the dataset. Finally, we got 971 congeneric series with an average of about 34 ligands per series.
2) For the performance analysis of PBCNet on FEP1 and FEP2 sets (Fig. 2A), the sample size for each analysis was determined by the maximum number of eligible samples available in the respective datasets (bin 0.0-0.2: n=18, bin 0.2-0.4: n=1567, bin 0.4-0.6: n=3071, bin 0.6-0.8: n=2404, bin 0.8-1.0: n=195).
3) For the results in 'The performance of PBCNet on ΔpIC_50 calculation' section, we all performed 10 independent runs with different random seed (n=10). Since our model requires a reference molecule, the choice of the reference molecule affects the performance of the model. In order to more fully validate the model performance, we conducted 10 independent experiments. The reason for setting n=10 is that there is one test series which contains only 11 molecules and n=10 is sufficient to make a full assessment of the model performance.
4) For the results in 'Using active learning in PBCNet to accelerate lead optimization ' section, we all performed 6 independent runs with different random seed(n=6). The process involves fine-tuning the model. Randomness in the AI model training process affects the training of the model, which is unavoidable. In this experiment we set up the random seed in order to control the randomness and ensure that the relevant experimental results can be reproduced.
5) In the model robustness validation experiments, the sample size is determined by the maximum number of ligand poses that can be produced by the docking software in a regular process (Bace: n=7, CDK2: n=3, JNK1: n=3, MCL1: n=5, p38: n=3, PTP1B: n=7, Thrombin: n=3, Tyk2: n=6).

Data exclusions

SMILES that failed during preparation with RDKit were removed. Binding affinity measurements without values as well as uncertain, i.e., qualified data with either the “<” or “>” sign, were discarded.

Replication

To reproduce the primary results of this research, refer to the analytical pipeline available at https://doi.org/10.5281/zenodo.8275244. All experimental results can be successfully reproduced.

Randomization

1) In the selection experiments, we initialized the model using random seeds 0 to 6. Randomness in the AI model training process affects the training of the model, which is unavoidable. In this experiment we set up the random seed in order to control the randomness and ensure that the relevant experimental results can be reproduced.
2) In the model ranking performance evaluation, we randomly conducted 10 independent runs in every experiment. Since our model requires a reference molecule, the choice of the reference molecule affects the performance of the model. In order to more fully validate the model performance, we conducted 10 independent experiments. The reason for setting n=10 is that there is one test series which contains only 11 molecules and n=10 is sufficient to make a full assessment of the model performance.

Blinding

We were blinded to the group allocation during data collection and analysis.The group allocation process was performed by computer script without any manual intervention.

Reporting for specific materials, systems and methods

We require information from authors about some types of materials, experimental systems and methods used in many studies. Here, indicate whether each material, system or method listed is relevant to your study. If you are not sure if a list item applies to your research, read the appropriate section before selecting a response.

Materials & experimental systems

n/a	Involved in the study
☒	☐ Antibodies
☒	☐ Eukaryotic cell lines
☒	☐ Palaeontology and archaeology
☒	☐ Animals and other organisms
☒	☐ Clinical data
☒	☐ Dual use research of concern

Methods

n/a	Involved in the study
☒	☐ ChIP-seq
☒	☐ Flow cytometry
☒	☐ MRI-based neuroimaging
